# Supplementary material for: An in vitro stem cell model of human epiblast and yolk sac interaction
Source: eLife. 2021 Aug 17;10:e63930. doi: 10.7554/eLife.63930 (PMC8370770; doi:10.7554/eLife.63930)
Supplement: Supplementary file 4. [file elife-63930-supp4.docx]

Supplementary Table S4:

| **Gene** | **Forward Primer 3’-5’** | **Reverse Primed 5’-3’** |
| --- | --- | --- |
| SOX2 | GAGCTTTGCAGGAAGTTTGC | GCAAGAAGCCTCTCCTTGAA |
| NANOG | GATTTGTGGGCCTGAAGAAA | CAGATCCATGGAGGAAGGAA |
| OCT4 | AGTGATTCTCCTGCCTCAGC | CTTCTGCTTCAGGAGCTTGG |
| GATA6 | GTTGGCACAGGACAATCCAAG | GTTGGCACAGGACAATCCAAG |
| EOMES | GTGCCCACGTCTACCTGTG | CCTGCCCTGTTTCGTAATGAT |
| SOX17 | TTCGTGTGCAAGCCTGAGAT | TAATATACCGCGGAGCTGGC |
| DKK1 | ATAGCACCTTGGATGGGTATTCC | CTGATGACCGGAGACAAACAG |
| GSC | GAGGAGAAAGTGGAGGTCTGGTT | CTCTGATGAGGACCGCTTCTG |
| MIXL1 | CCGAGTCCAGGATCCAGGTA | CTCTGACGCCGAGACTTGG |
| FOXA2 | CATGCCTGGCAGCTTGG | CTCTCTCACTTGTCCTCGATCC |
| CER1 | ACAGTGCCCTTCA-GCCAGACT | ACAACTACTTTTTCACAGCCTTCGT |
| NOG | CCATGCCGAGCGAGATCAAA | TCGGAAATGATGGGGTACTGG |
| T | AGCCAAAGACAATCAGCAGAAA | CACAAAAGGAGGGGCTTCACTA |
| NESTIN | AGCGTTGGAACAGAGGTTG | AGGAGGGTCCTGTACGTG |
| NOTCH1 | ATCCTGATCCGGAACCGAG | CGTCGTGCCATCATGCAT |
| PAX6 | CTGAGGAATCAGAGAAGACAGGC | ATGGAGCCAGATGTGAAGGAGG |
| HRPT | TGACACTGGCAAAACAATGCA | GGTCCTTTTCACCAGCAAGCT |

Supplementary Table S5:

| **Primary Antibody** | **Source** | **Dilution** |
| --- | --- | --- |
| DKK1 | R&D Systems, AF1096-SP | 1:200 |
| CER1 | R&D Systems, AF1075-SP | 1:200 |
| SOX2 | Abcam, Ab59776 | 1:200 |
| GATA6 | R&D Systems, AF1700 | 1:200 |
| pSMAD1/5 | Cell Signalling Technology, 13820S | 1:200 |
| LEF1 | Cell Signalling Technology, 2230S | 1:200 |
| T | R&D Systems, AF2085 | 1:200 |
| SOX1 | R&D Systems, AF3369-SP | 1:200 |
| OTX2 | R&D Systems, AF1979-SP | 1:200 |
| NANOG | R&D Systems, AF1997 | 1:200 |
| Human nuclear antigen | Abcam, ab191181 | 1:200 |

Supplementary table S6:

| **Secondary Antibody** | **Source** | **Dilution** |
| --- | --- | --- |
| MOUSE 488 | Invitrogen, A21202 | 1:500 |
| RABBIT 568 | Invitrogen, A10042 | 1:500 |
| GOAT 647 | Invitrogen, A21447 | 1:500 |
| GOAT 594 | Invitrogen, A11057 | 1:500 |
| PHALLOIDIN 647 | Invitrogen, A30104 | 1:200 |
| DAPI | Invitrogen, D3571 | 1:500 |
